# Two-dimensional segmentation fusion tool: an extensible, free-to-use, user-friendly tool for combining different bidimensional segmentations

**DOI:** 10.3389/fbioe.2024.1339723

**Published:** 2024-01-31

**Authors:** Filippo Piccinini, Lorenzo Drudi, Jae-Chul Pyun, Misu Lee, Bongseop Kwak, Bosung Ku, Antonella Carbonaro, Giovanni Martinelli, Gastone Castellani

**Affiliations:** ^1^ IRCCS Istituto Romagnolo per lo Studio dei Tumori (IRST) “Dino Amadori”, Meldola, Italy; ^2^ Department of Medical and Surgical Sciences (DIMEC), University of Bologna, Bologna, Italy; ^3^ Student, Computer Science and Engineering, University of Bologna, Bologna, Italy; ^4^ Department of Materials Science and Engineering, Yonsei University, Seoul, Republic of Korea; ^5^ Division of Life Sciences, College of Life Science and Bioengineering, Incheon National University, Incheon, Republic of Korea; ^6^ Institute for New Drug Development, College of Life Science and Bioengineering, Incheon National University, Incheon, Republic of Korea; ^7^ College of Medicine, Dongguk University, Goyang-si, Gyeonggi-do, Republic of Korea; ^8^ Central R&D Center, Medical and Bio Decision (MBD) Co., Ltd., Suwon, Republic of Korea; ^9^ Department of Computer Science and Engineering (DISI), University of Bologna, Cesena, Italy

**Keywords:** histology, oncology, microscopy, segmentation, image fusion, free-to-use tool, standalone application

## Abstract

**Introduction:** In several fields, the process of fusing multiple two-dimensional (2D) closed lines is an important step. For instance, this is fundamental in histology and oncology in general. The treatment of a tumor consists of numerous steps and activities. Among them, segmenting the cancer area, that is, the correct identification of its spatial location by the segmentation technique, is one of the most important and at the same time complex and delicate steps. The difficulty in deriving reliable segmentations stems from the lack of a standard for identifying the edges and surrounding tissues of the tumor area. For this reason, the entire process is affected by considerable subjectivity. Given a tumor image, different practitioners can associate different segmentations with it, and the diagnoses produced may differ. Moreover, experimental data show that the analysis of the same area by the same physician at two separate timepoints may result in different lines being produced. Accordingly, it is challenging to establish which contour line is the ground truth.

**Methods:** Starting from multiple segmentations related to the same tumor, statistical metrics and computational procedures could be exploited to combine them for determining the most reliable contour line. In particular, numerous algorithms have been developed over time for this procedure, but none of them is validated yet. Accordingly, in this field, there is no ground truth, and research is still active.

**Results:** In this work, we developed the *Two-Dimensional Segmentation Fusion Tool* (*TDSFT*), a user-friendly tool distributed as a free-to-use standalone application for *MAC*, *Linux*, and *Windows*, which offers a simple and extensible interface where numerous algorithms are proposed to “compute the mean” (i.e., the process to fuse, combine, and “average”) multiple 2D lines.

**Conclusions:** The *TDSFT* can support medical specialists, but it can also be used in other fields where it is required to combine 2D close lines. In addition, the *TDSFT* is designed to be easily extended with new algorithms thanks to a dedicated graphical interface for configuring new parameters. The *TDSFT* can be downloaded from the following link: https://sourceforge.net/p/tdsft.

## 1 Introduction

Contouring refers to the outlining of specific structures or areas in a target image. It entails defining the border of the foreground region (i.e., object of interest) and highlighting this border as a closed line of 1-pixel size in a 2D binary mask (i.e., black and white images). On the other hand, segmentation involves the definition of the foreground followed by the highlighting of this region with a dense surface, allowing the precise identification of a group of specific pixels (Hemalatha et al., 2018). This is a common image preprocessing step in several fields, ranging from autonomous driving, where vehicles, pedestrians, and road markings are segmented for detection and tracking purposes (Xiao et al., 2023), to agriculture/geology, where it is used to identify and classify different crops (Shoaib et al., 2022) or analyze soil conditions (Rippner et al., 2022). In the medical field, segmenting objects of interest is a widespread step, particularly in several fields of oncology (Jiménez and Racoceanu, 2019). For instance, in the context of radiotherapy, this involves delineating tumor volumes or areas at risk of microscopic disease, as well as normal anatomical structures, such as organs at risk. The goal of segmenting is to accurately define these structures to guide the radiation treatment process and ensure optimal patient outcomes (Lin et al., 2020). Similarly, segmenting objects is a very popular task in histology where the target is different, typically a microscopy image, but the main goal is to always spatially define the pixels belonging to different tissue regions for proceeding with further analysis (Bocchini et al., 2023).

**FIGURE 1:**
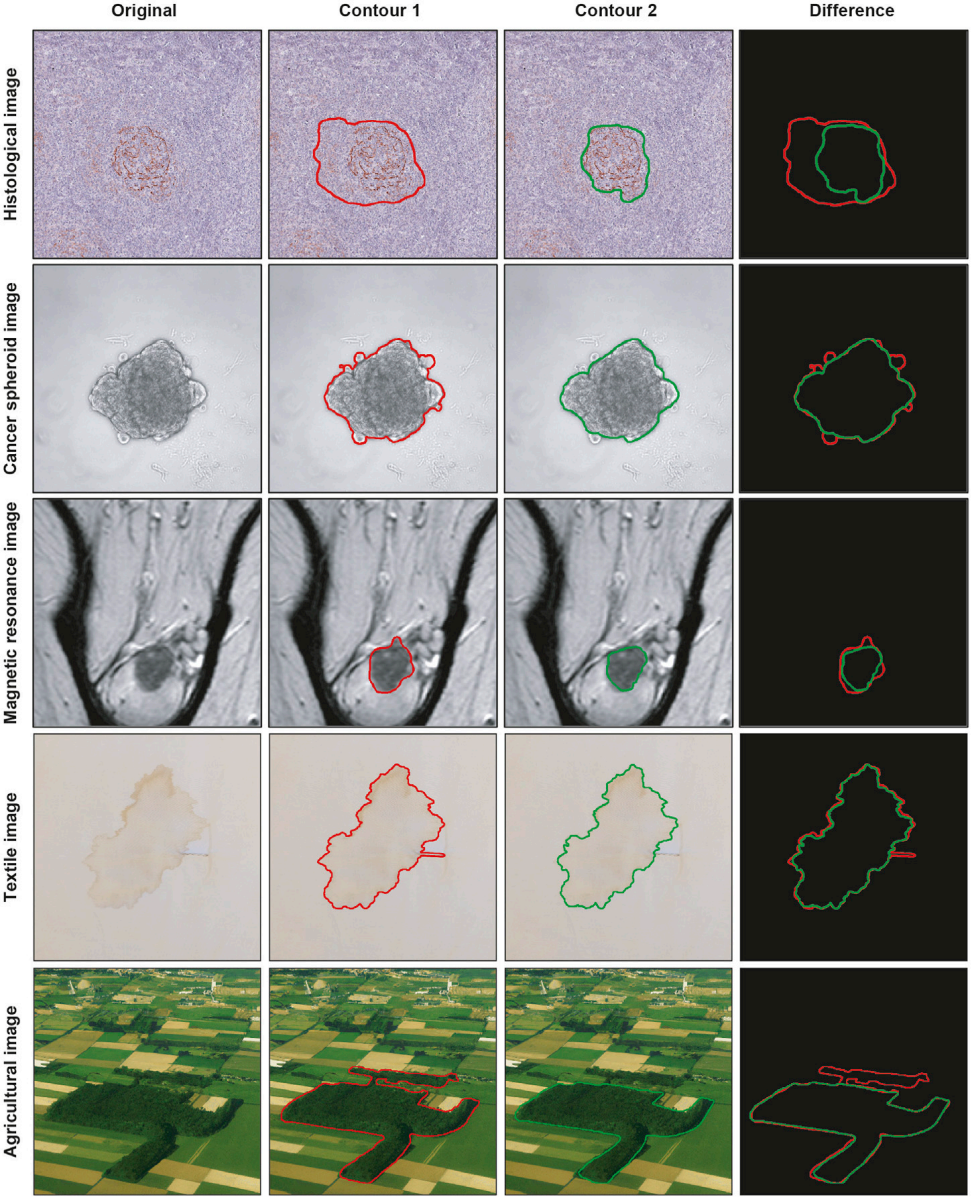
Example of segmentations obtained by two different practitioners. From top to bottom: histological image, cancer spheroid image, magnetic resonance image, textile image, and agricultural image. From left to right: original image, contour obtained from the first annotator, contour obtained from the second annotator, and overlaid contours.

Segmenting a cancer area is a time-consuming and labor-intensive task. It requires significant effort from practitioners, mainly medical doctors and physicians involved in cancer treatment planning (Hu et al., 2012), spending every day several hours of their working time in segmenting images. Typically, they use manual annotation tools [e.g., *ImageJ; ROI Manager* (Schneider et al., 2012) and *AnaSP* (Piccinini, 2015)] and computer-aided design (CAD) systems [e.g., *MITK* (Tasnadi et al., 2020a) and *QuPath* (Bankhead et al., 2017)]. However, in general, there are inter- (i.e., differences between segmentations created by different practitioners, also known as reproducibility) and intra-rater reliability (i.e., differences between segmentations created by the same practitioner but at different times, also known as repeatability) issues (Piccinini et al., 2017), and defining the correct segmentation (i.e., ground truth) is challenging. Accordingly, several statistical metrics and computational procedures are typically involved to determine the border of the object/area of interest in the image (Ramesh et al., 2021). These range from threshold-based segmentation approaches (Bai and Zhou, 2023) to deep-learning ones (Piccinini et al., 2023), never excluding the manual segmentation, which is still the gold standard for several applications (Veta et al., 2013). However, more 2D segmentation masks (typically represented as binary masks of the same size of the input image, with the object of interest identified by a white region on a black background) are available because they are (*a*) obtained in different ways, (*b*) or by different practitioners, (*c*) or by the same practitioner but at different times. Figure 1 shows the example of segmentations obtained by two different annotators, analyzing a slide scanner histological image, a microscopy cancer spheroid image, a magnetic resonance slide, a textile photograph, and an agricultural picture. In these cases, fusion algorithms are typically used for “fusing” (i.e., a process for combining, averaging, and “computing the mean”) multiple 2D segmentations. Nevertheless, nowadays, there is no standard for this procedure, and different fusing solutions have been proposed in the literature (James and Dasarathy, 2014).

In this work, in addition to reviewing all the fusion algorithms proposed in the literature for averaging different 2D segmentations, we developed the *Two-Dimensional Segmentation Fusion Tool* (*TDSFT*), an extensible, user-friendly *MATLAB* (i.e., Matrix Laboratory) tool collecting more fusion algorithms. Free-to-use standalone versions have been provided for *MAC*, *Linux*, and *Windows*, and the *MATLAB* source code can be openly read. A commercial license of MATLAB is needed just in case the user wants to modify the code. The *TDSFT* simply requires a series of 2D binary segmentations as input (i.e., binary masks of the same size of the input image, with the object of interest identified by a dense white region on a black background) and provides a new binary mask with a white one-size pixel closed line of the foreground’s contour as output, computed according to the fusion algorithm and closing method selected. Therefore, the *TDSFT* can support medical specialists, but it can also be used in other fields where it is required to combine 2D closed lines.

The current version of *TDSFT* (i.e., version 1) offers eight different fusion algorithms to average multiple 2D segmentations and four fitting/interpolating methods for closing eventually sparse 2D lines. In addition, the *TDSFT* is designed to be easily extended with new fusion algorithms thanks to a dedicated graphical user interface (GUI) for configuring new parameters. A *TDSFT* source code, free-to-use standalone applications for *MAC*, *Linux*, and *Windows*, video tutorial, documentation, and sample datasets are available at the following link: https://sourceforge.net/p/tdsft.

## 2 Methods

In the next sections, the *TDSFT*’s structure, the four available fitting/interpolating methods, and the eight implemented fusion algorithms are described in detail.

### 2.1 Two-dimensional segmentation fusion tool—Backbone

**FIGURE 2:**
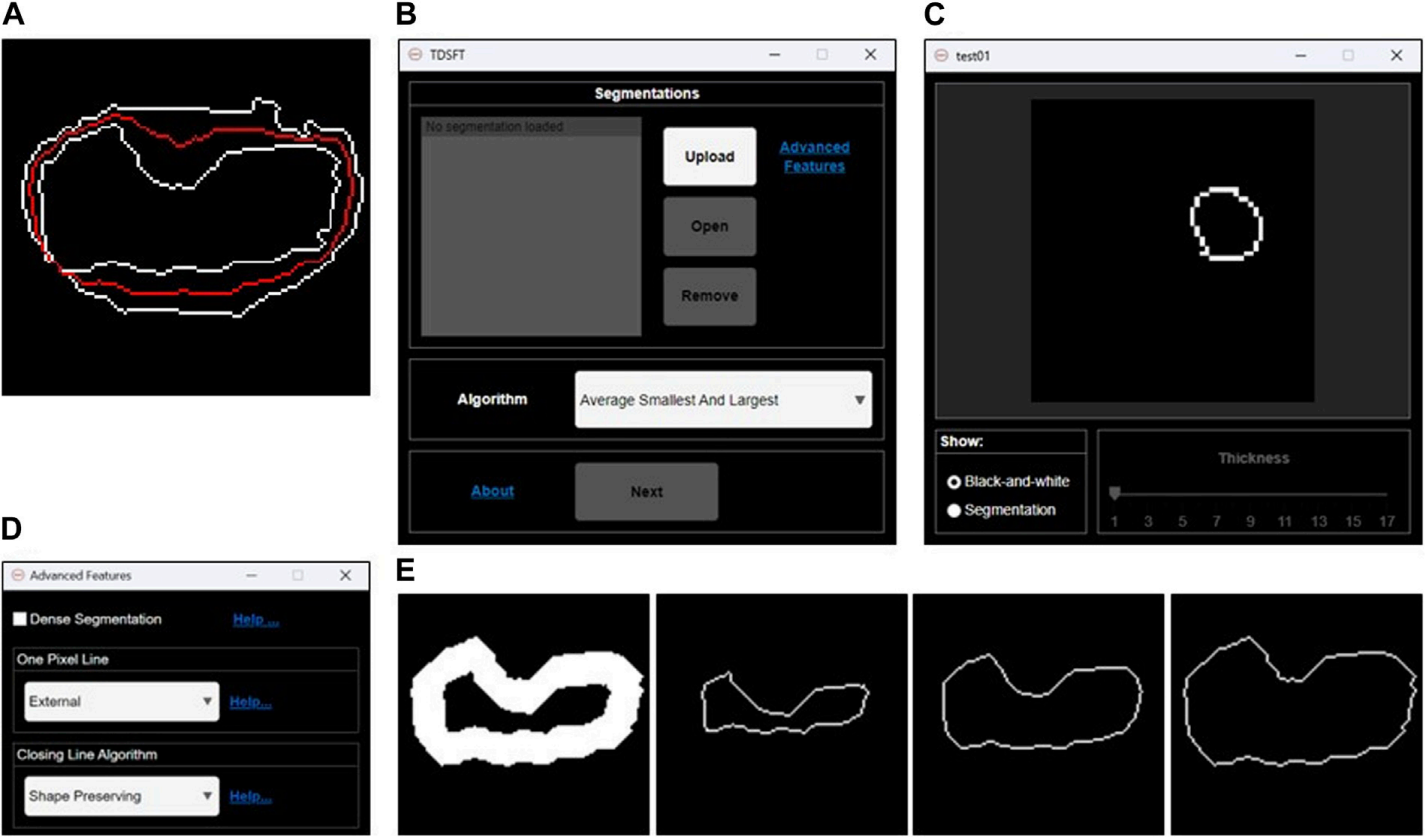
Fusion of 2D segmentations. **(A)** Example of bidimensional segmentation fusion using the algorithm named “*Average Smallest and Largest*.” **(B)** Main graphical user interface for loading and managing the 2D segmentations and selecting the fusion algorithm to be used. **(C)** Preview GUI for analyzing the single 2D segmentations. **(D)** Advanced feature GUI for optional parameters. **(E)** Examples of 1-pixel lines extracted by an original 2D segmentation larger than one pixel. From left to right: internal 1-pixel line, middle skeletonized 1-pixel line, and external 1-pixel line.

The *TDSFT* is an extensible, free-to-use, user-friendly tool that offers several algorithms for the fusion of multiple bidimensional segmentations (Figure 2A). The *TDSFT* is developed using *MATLAB R2022b* as a modular and organized structure project developed using the model-view-controller (MVC, Supplementary File 1) pattern, strongly helping for extension. Accordingly, in case of literature published on new reliable fusion algorithms, it will be easy to include them in the tool. To be able to use the software without a license, the *TDSFT* is also available as a standalone application for *Windows*, *Linux*, and *MAC*.

**FIGURE 3:**
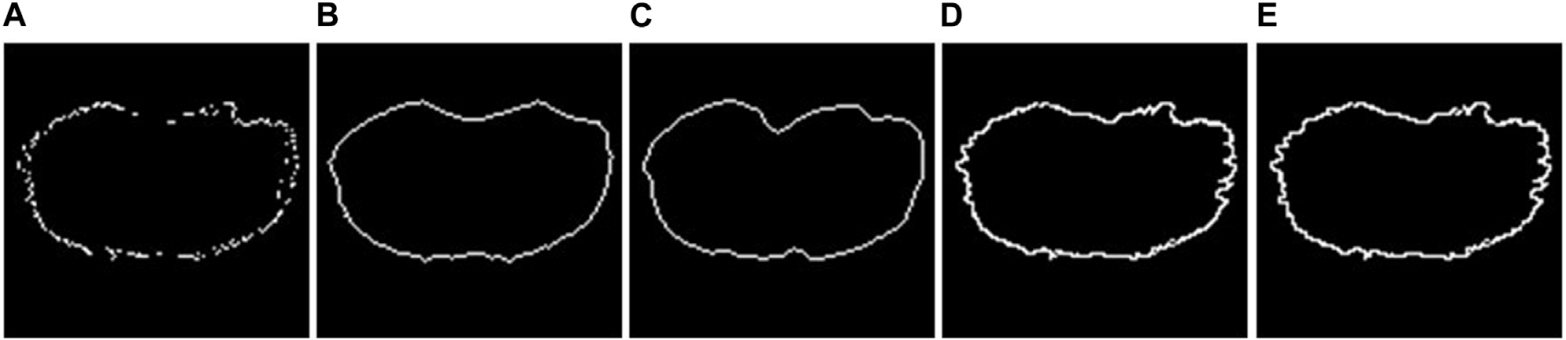
2D fitting methods. **(A)** Example of an unclosed 2D segmentation. Example of results obtained using **(B)** Chan–Vese fitting method; **(C)** Geodesic Active Contour fitting method; **(D)** linear-based interpolation method; **(E)** shape-preserving-based interpolation method.

The *TDSFT*’s input is composed of a series of binary segmentations (several image formats are supported, including the classical *tif*, *bmp*, and *png*), which is managed through the main GUI with the “*Upload*,” “*Open*,” and “*Remove*” buttons (Figure 2B). In particular, the single-uploaded segmentations can be analyzed in a separate GUI that is automatically visualized by clicking the “*Open*” button (Figure 2C). The users can then choose the algorithm to be used for the fusion process from the drop-down menu named “*Algorithm*.” Using the “*Advanced Features*” link, it is also possible to open the advanced feature window (Figure 2D) for deciding (*a*) how to compute the one-pixel segmentations in case of original segmentations composed by a line with a diameter of more than one pixel (the possible solutions are an “*internal*,” “*middle*,” or “*external*” line, Figure 2E); (*b*) the algorithm to be used just for closing possibly unclosed 1-pixel size contour lines that can be obtained as output from several fusion algorithms (Figure 3).

The closing methods offered can be divided into two groups: (*I*) *Active contour methods*: These methods iteratively adjust a contour’s position to align with object edges by minimizing an energy function that combines image and shape information. Not all the pixels of the original unclosed segmentation are preserved. (*II*) *Interpolation methods*: All the pixels of the original unclosed segmentation are preserved, and each pixel must be connected to at least two other points for obtaining a final closed line. The biggest issue, however, is to identify neighboring points for each pixel. In many circumstances, it can be difficult to find them in the Cartesian reference system. Because of this, the implementation option is to convert them to the polar reference point system and translate all coordinates around the largest segmentation’s center point. This enables them to order the points according to their angular position and use that information to identify their neighbors. Finally, a closed segmentation can be achieved by applying an interpolation method, creating a line between the pixels.

The algorithms belonging to the active contour group are implemented using built-in *MATLAB* functions as follows: (*I1*) *Chan–Vese* (Chan and Vese, 2001): It is designed to segment objects without clearly defined boundaries. This method relies on iteratively evolving sets of levels to minimize a multi-term function called energy. (*I2*) *Geodesic* (Caselles et al., 1997): It is based on active contours evolving in time according to intrinsic geometric measures of the image.

Instead, the interpolation methods are implemented by us, and they can also be found as external specific functions officially uploaded to the *MathWorks file exchange* website at the link: https://it.mathworks.com/matlabcentral/fileexchange/134951-closing-2d-line-with-interpolation. (*II1*) *Linear interpolation*: Each pair of adjacent points is connected by a segment that can be calculated independently of the others. If we denote (*x*_*i*_, *y*_*i*_) and (*x*_*i+1*_, *y*_*i+1*_) as the pair of adjacent points, the interpolating function *f*_*i*_(*x*) is defined as reported in Eq. 1:

$$f_{i}\left(x\right)=\frac{x_{i+1}-x}{x_{i+1}-x_{i}}{\;y}_{i}+\frac{x-x_{i}}{x_{i+1}-x_{i}}\;y_{i+1}.$$ (1)

(II2) *Piecewise cubic Hermite interpolation* (*PCHIP*, *shape-preserving*) (Fritsch and Carlson, 1980): *PCHIP* interpolates using a piecewise cubic polynomial with these properties: (*a*) on each subinterval, the polynomial *P*(*x*) is a cubic Hermite interpolating polynomial for the given data points with specified derivatives at the interpolation points. (*b*) *P*(*x*) interpolates *y*, that is, *P*(*x*_*j*_) = *y*_*j*_, and the first derivative is continuous. The second derivative is probably not continuous, so jumps are possible. (*c*) The cubic interpolant *P*(*x*) is shape-preserving. The slopes at the *x*_*j*_ are chosen in such a way that *P*(*x*) preserves the shape of the data and respects monotonicity.

Furthermore, the *TDSFT* is extensible. Users can add and execute their fusion algorithms and closing methods. The process is supported by the documentation and the video tutorial. Furthermore, the user can set up a dedicated *GUI* for runtime parameters by just using a simple *JSON* file.

### 2.2 Implemented fusion algorithms—description

**FIGURE 4:**
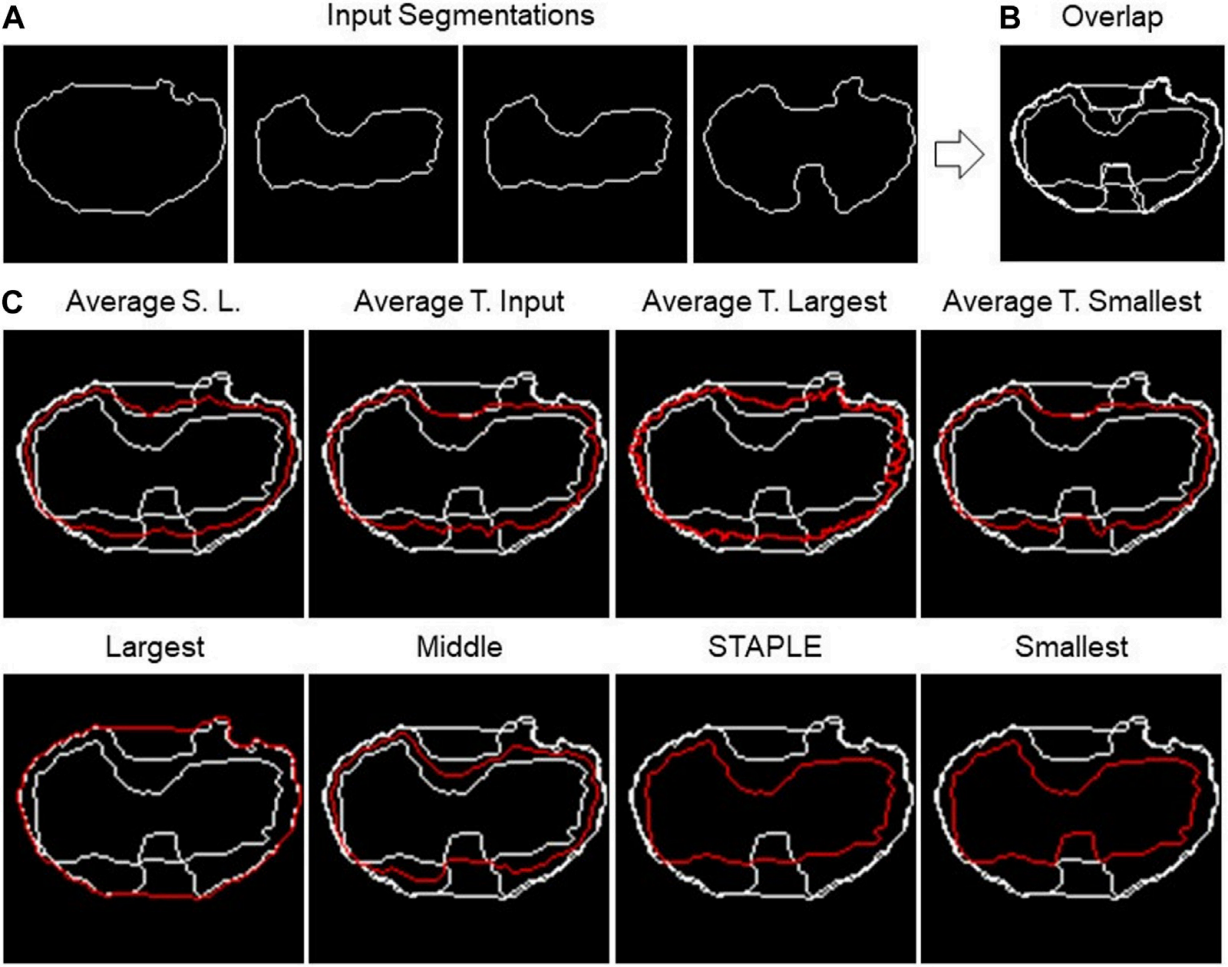
2D fusion algorithms. **(A)** Example of four different input segmentations, one each panel and then **(B)** overlapped. **(C)** Example of results obtained using eight different fusion algorithms (the names are reported above the single panels) using the shape-preserving-based interpolation method when needed.

The *TDSFT* offers eight different fusion algorithms. In the next paragraphs, they are also described by exploiting the examples reported in Figure 4, showing the different algorithms’ output using the same input segmentation (Figure 4A). In addition, Supplementary File 2 reports flowcharts describing all their steps.

The first algorithm to be presented is named as “*Largest*,” which calculates the segmentation containing all the input segmentations. Basically, it is the contour of the “*Union*” of the different segmentations. The process consists of a few steps. As a first step, all segmentations are overlapped, then the hole-filling operation is performed, and finally, the perimeter of the resulting area is calculated.

The opposite algorithm to the previous one is called “*Smallest*,” which calculates the perimeter of the area shared by all segmentations. Essentially, it is the contour of the “*Intersection*” of the different segmentations. In this instance, the hole-filling procedure is carried out on all segmentations prior to completing the overlap. Then, appropriate matrices are then added to achieve their overlap. The area whose pixel value is equal to the number of segmentations is now referred to as the common area.

On the other hand, “*Average Smallest And Largest*” calculates the 1-pixel segmentation between the *Smallest* and the *Largest* segmentation. The *Smallest* and *Largest* segmentation are first calculated and are then overlapped. Finally, the skeletonization operation is applied to obtain the 1-pixel middle line.

The algorithms “*Average Target Largest*,” “*Average Target Smallest*,” and “*Average Target From Input*” are part of the same family of algorithms, with the only difference in the choice of target segmentation, the one from which the computation starts. In the first two, *Largest* and *Smallest* are used, and in the third, the user can choose the target between one of the input segmentations. After choosing the target segmentation, the process is the same for all these algorithms. Given a set of input segmentations and a target segmentation, the algorithm iterates over each pixel of the latter and for each of these computes the new pixel of the average segmentation. The process of obtaining a new pixel can be divided into two steps: (*1*) the closest pixel of any other segmentation to the chosen target segmentation’s pixel is calculated in order to create a group of points; (*2*) given the points obtained in step *1*, the new pixel is calculated as follows: (*a*) if the points are two, the midpoint of the segment having these as extremes is calculated; (*b*) if the points are more than two but belong to the same line (collinear points), the two extremes are calculated, and the midpoint of the segment is calculated; (*c*) in other cases, the centroid of the polygon described by the set of points is calculated.

The next algorithm to be discussed is called “*Middle*,” which calculates the segmentation in the center of all the input ones. The first step is to overlap all the segmentations. Then, the algorithm operates in successive steps, and at each iteration, the smallest and the largest segmentation are removed. The total number of iterations is defined as the lower bound (i.e., floor integer) of (*N.Seg.* −1)/2, with *N.Seg*., and the number of available segmentations. At the end of the iterations, there are two possible situations: (*1*) if the number of input segmentations is odd, and then at the end of the iterations, only one segmentation will remain, which will then be the central segmentation; (*2*) if the number of input segmentations is even, on the other hand, there are two remaining segmentations. To obtain the result, the practitioner must specify the algorithm to be used to fuse the last two segmentations. The algorithms offered for this purpose are *Average Smallest and Largest*, *Largest*, and *Smallest*.

The last algorithm is “*Simultaneous Truth and Performance Level Estimation (STAPLE)*” (Warfield et al., 2004). *STAPLE* is a weighted voting algorithm that takes into account all segmentations while computing the outcome. As an initial step, the algorithm will combine all the segmentations into a test segmentation by simply voting on each pixel. *STAPLE* will rate each annotator’s accuracy in relation to this initial test segmentation. Then, it will redraw a new segmentation by weighting the votes of the specialists according to their accuracy. Because *STAPLE* is iterative, this cycle of estimating the accuracy and redrawing the test segmentation will repeat until the test segmentation stops changing or the maximum number of iterations is reached. The final test segmentation will be the “*ground truth*” that *STAPLE* returns. The implementation used in the *TDSFT* can be found at the following link: https://www.mathworks.com/matlabcentral/fileexchange/56789-staple-d.

## 3 Experiments

Comparing the different fusion algorithms and defining which is the best one is really challenging because there is not a general best one, and the better one depends on several factors (e.g., the presence of outlier segmentations and availability of many similar segmentations making the dataset unbalanced). This is the reason why we provided the user of several algorithms and not just a “winning” one. However, to provide a proof of concept on how the algorithms perform, we selected a representative case of study, a microscopy dataset that is publicly available, composed by different segmentations, obtained using freely available tools, and a manual ground truth. In the next sections, the experimental setup, the used metric, and the results obtained are described in detail.

### 3.1 Experimental setup—description

To analyze the performances of the different fusion algorithms, we used a dataset related to a cancer multicellular spheroid, imaged with a light-sheet fluorescence microscope (LSFM) (Stelzer et al., 2021). Tasnadi et al. (2020b) already used this dataset, testing several segmentation algorithms and disclosing all their specifics (which are not relevant to our research in this case). The spheroid is composed of 52 cells, and for each cell, the dataset includes two manual segmentations, one created by an expert microscopist operator and one created by a microscopist researcher, with a limited number of years of experience and five different segmentations automatically obtained using different freely available tools, precisely *3D-Cell-Annotator* (*3DCA*) (Tasnadi et al., 2020b), *MINS* (Lou et al., 2014), *Pagita* (Gul-Mohammed et al., 2014), *XPIWIT* (Bartschat et al., 2016), and *OpenSegSpim* (Gole et al., 2016).

The images of the multicellular spheroid are 3D, precisely a 3D stack with each image representing a different z section of the spheroid. However, the *TDSFT* accepts as input binary two-dimensional (2D) segmentations. Accordingly, the procedure applied to create the testbed for the experiments are as follows: (*a*) 10 different sections from the 3D stack were randomly selected; (*b*) for each section, a cell was randomly chosen; (*c*) for each selected cell and selected section, the manual segmentation created by the expert microscopist was considered the ground truth, and the six other segmentations (i.e., *second human annotator*, *3DCA*, *MINS*, *Pagita*, *XPIWIT*, and *OpenSegSpim*) were used for testing the different fusion algorithms. The configuration set for the advanced features was the default one, basically with the parameters “*External one-pixel line*” for pre-processing the input binary 2D segmentations, and “*Shape-preserving*” as the closing method in case of sparse pixels. Furthermore, specifically for the algorithm “*Average Target From Input*,” the target segmentation selected was the one obtained using *3DCA*.

The dataset used in the experiments of this work is publicly available for further analysis at the following link: https://sourceforge.net/p/tdsft.

### 3.2 Performance metric—description

The most appropriate way to carry out the comparison of segmentations is so far unclear (Warfield et al., 2004). In the literature, several metrics have been proposed to compare segmentations. Simply measuring the volume of segmented structures (Iosifescu et al., 1997; Warfield et al., 2000) or assessing the limits of agreement (Bland and Altman, 2003) of volume estimates derived from the segmentations is something common. However, measures of spatial overlap are the metrics most widely applied (Dice, 1945). Alternative metrics have been sought (Everingham et al., 2002). For instance, in many applications, assessment of boundary differences is useful, and the Hausdorff measure and modifications have been used (Gerig et al., 2001). In addition, agreement measures, such as the kappa statistic, have also been explored (Zijdenbos et al., 1994). In conclusion, nowadays, there is not a single globally used metric for this purpose.

In our case, to compare the different fusion algorithms and evaluate the results obtained, we decided for the *Jaccard index* (JI), also known as Intersection over Union (IoU) or the Jaccard similarity coefficient (Piccinini et al., 2020). It is a well-known metric used for evaluating the similarity of two sample sets (e.g., A and B). JI (A and B) is mathematically defined as the size of the intersection (i.e., |A ∩ B|, the number of overlapping voxels) divided by the size of the union (i.e., |A ∪ B|) of the sample sets, according to Eq. 2:

$$\text{JI }\left(A,B\right)=\left|A\cap B\right|/\left|A\cup B\right|=\left|A\cap B\right|/\left(\left|A\right|+\left|B\right|-\;\left|A\cap B\right|\right).$$ (2)

### 3.3 Results

Table 1 reports the JI values obtained by analyzing the spheroid dataset (composed by the 10 different sections of the cells randomly selected, hereafter named *Cell#*, with *#* ranging from *1* to *10*), and comparing with the ground truth the result of the different versions of the fusion algorithms. Precisely, the 10 different versions of the fusion algorithms compared are

1. *Average Smallest And Largest.*
2. *Average Target From Input (3DCA)*, with the segmentation obtained using *3DCA* as the target line.
3. *Average Target Largest*, with the segmentation obtained with the “*Largest*” algorithm used as the target line.
4. *Average Target Smallest*, with the segmentation obtained with the “*Smallest*” algorithm used as the target line.
5. *Largest.*
6. *Middle—Average*, with the final line computed using the “*Average*” algorithm in case of an even number of input segmentations.
7. *Middle—Largest*, with the final line computed using the “*Largest*” algorithm in case of an even number of input segmentations.
8. *Middle—Smallest*, with the final line computed using the “*Smallest*” algorithm in case of an even number of input segmentations.
9. *Smallest.*
10. *STAPLE.*

**TABLE 1:** Jaccard indexes of the fused segmentations obtained with the different algorithms.

Algorithm	Cell 1	Cell 2	Cell 3	Cell 4	Cell 5	Cell 6	Cell 7	Cell 8	Cell 9	Cell 10	Average
*Average Smallest And Largest*	0.8768	0.9448	0.8667	0.9064	0.8226	0.8775	0.9023	0.9586	0.9067	0.8456	0.8908
*Average Target From Input (3DCA)*	0.8045	0.8792	0.8598	0.8475	0.8707	0.8719	0.8902	0.9231	0.7815	0.8406	0.8569
*Average Target Largest*	0.8318	0.9013	0.8571	0.8238	0.8547	0.8529	0.9146	0.8970	0.7852	0.8529	0.8571
*Average Target Smallest*	0.7972	0.8733	0.7152	0.9064	0.8211	0.8366	0.9064	0.8953	0.7933	0.7226	0.8268
*Largest*	0.8782	0.9045	0.8457	0.8240	0.6538	0.9120	0.8791	0.8859	0.8171	0.7485	0.8349
*Middle—Average*	0.8619	0.8690	0.8466	0.9067	0.8654	0.8081	0.8875	0.8650	0.6846	0.8692	0.8464
*Middle—Largest*	0.8619	0.8690	0.8466	0.9067	0.8846	0.8010	0.8875	0.8650	0.6846	0.8692	0.8466
*Middle—Smallest*	0.8190	0.7862	0.8037	0.8497	0.8333	0.7761	0.8688	0.8466	0.6309	0.7891	0.8003
*Smallest*	0.5238	0.7103	0.4969	0.6354	0.6765	0.6070	0.7813	0.7423	0.5101	0.5156	0.6199
*STAPLE*	0.8826	0.9178	0.8698	0.9124	0.8333	0.8458	0.9000	0.8841	0.6846	0.8846	0.8615

Table 2 reports the rank’s position of the 10 different versions of the tested fusion algorithms, according to the average value of the JI (i.e., Table 1, last column) computed by considering together the 10 different cells. Despite the absolute values being dataset-dependent, it is possible to observe general results just by considering the rank positions of the fusion algorithms.

**TABLE 2:** Final rank’s positions of different fusion algorithms.

Rank’s position	Algorithm	Average
1/10	*Average Smallest And Largest*	0.8908
2/10	*STAPLE*	0.8615
3/10	*Average Target Largest*	0.8571
4/10	*Average Target From Input (3DCA)*	0.8569
5/10	*Middle—Largest*	0.8466
6/10	*Middle—Average*	0.8464
7/10	*Largest*	0.8349
8/10	*Average Target Smallest*	0.8268
9/10	*Middle—Smallest*	0.8003
10/10	*Smallest*	0.6199

First of all, it is worth considering that in a set of data, the outer and the inner lines are most of the time noisy instances, called outliers. Translating this general concept to the segmentations, the *Smallest* and *Largest* algorithms, based on the definition of the outer and inner lines, are outlier-dependent and, in fact, are characterized by some of the worst performances (position 10/10 and 7/10 in the rank, respectively). All the algorithms based on the *Average* of the different segmentations (defined by considering a specific line as a target) obtained similar values, which were all reported in the central part of the ranking (rank’s positions 8/10, 4/10, and 3/10). Similarly, all the algorithms based on the selection as output of the line in the *Middle* obtained similar results with excellent absolute JI values, except for the algorithm *Middle—Smallest* (rank’s position 9/10) selecting the smallest line in the middle, in case of an even number of segmentations (like in this experiment where we used the fusion algorithms as input of six different segmentations). However, there is no reliable explanation for a so different result obtained by the *Middle—Smallest* (i.e., 9/10) algorithm in comparison to the *Middle—Largest* one (i.e., 5/10). Finally, the best results were obtained using *STAPLE* (rank’s position 2/10) and *Average Smallest And Largest* (rank’s position 1/10). However, *STAPLE*, considering the contribution of all the input segmentations, for minimizing the potentially large uncertainty on the values of the estimated parameters, is suggested in case of a high number of input segmentations to be fused (Commowick and Warfield, 2010), while *Average Smallest And Largest*, being based just on the outer and inner lines, is suggested in case of a limited number of segmentations to be fused. For instance, in the practical case of just two input segmentations (one of the most recurrent cases in real scenarios), *Average Smallest And Largest* would be preferred to *STAPLE* (absolutely not recommended in this scenario because it is not designed for working with just two segmentations). Nevertheless, *Average Smallest And Largest*, being based on the *Largest* and *Smallest* algorithms, is noise-sensitive. Accordingly, for filtering the outliers and being more robust, our general suggestion is the *Middle—Average* (i.e., 6/10) algorithm that selects the line in the middle, without interpolating in the case of an odd number of input segmentations and exploiting the *Average* algorithm in the case of an even number of inputs. In addition, it is worth noting that *Average Smallest And Largest* (i.e., 1/10), and *Middle—Average* (i.e., 6/10) give precisely the same output in the case of just two input segmentations.

## 4 Conclusion

Segmenting objects of interest, specifically the segmentation of tumor areas in medical images, is a crucial and challenging step in various fields, including oncology and histology. The accuracy of segmentation is pivotal in guiding further decisions, but the lack of a standard for identifying object boundaries introduces subjectivity and variability into the process.

The subjective nature of the segmentation is evident as different operators can produce varying segmentations for the same tumor image. Moreover, even the same operator may produce different segmentations when analyzing the same area at different times.

To solve the difficulty of fusing multiple 2D segmentations to determine a reliable foreground’s contour line, many algorithms have been developed. However, none of these algorithms has achieved validation or standardization, leaving an ongoing research gap in this field.

In this paper, we are interested in different fusion algorithms (not segmentation ones). In particular, this study introduces the *TDSFT*, a free-to-use, user-friendly tool developed to facilitate the fusion of multiple 2D segmentations. Precisely, we implemented different fusion algorithms and compared them using publicly available datasets composed by different segmentations previously obtained with segmentation tools already published and validated. It is worth noting that the *TDSFT* is not limited to medical applications but can be employed in any field requiring the combination of 2D closed lines. It provides an interface for users to choose from multiple fusion algorithms and offers flexibility for adding new algorithms through a graphical interface.

To evaluate the performance of the fusion algorithms, experiments were conducted using a dataset of multicellular spheroid images, JI as a similarity metric, and 10 different fusion algorithms. The *Average Smallest And Largest* and *STAPLE* algorithms showed promising results, with their suitability depending on factors like the number of input segmentations. However, the *Middle—Average* algorithm was the one finally suggested because it is a robust choice for filtering outliers and producing reliable fusion results.

In conclusion, this study contributes to the ongoing efforts to address the subjectivity and variability in object segmentation by describing various fusion algorithms and introducing the free-to-use, user-friendly *TDSFT* tool. As future work, exploiting the extensibility of the *TDSFT*, we would like to implement and test a new algorithm first by discarding the *Largest* and *Smallest* lines for removing probable outliers and then applying the *Average Target From Input*, by considering the line obtained by previously applying the *Middle—Average* algorithm as the target input. This combination would balance the values from all the input lines (except the outer and inner ones) and would be a good solution for filtering noise when there are at least three input segmentations. In addition, we would like to include an optimized procedure for a parallel analysis of multiple objects.

The *TDSFT* source code, standalone application for *MAC*, *Linux*, and *Windows*, video tutorial, documentation, and sample datasets can be downloaded from the following link: https://sourceforge.net/p/tdsft.

## Data Availability

The datasets presented in this study can be found in online repositories. The names of the repository/repositories and accession number(s) can be found at: https://sourceforge.net/p/tdsft.

## Supplementary material

Supplementary Data Sheet 1 TDSFT MVC architecture.

Supplementary Data Sheet 2 Flowcharts of the different algorithms.
