# Supplementary material for: Two-dimensional segmentation fusion tool: an extensible, free-to-use, user-friendly tool for combining different bidimensional segmentations
Source: Front Bioeng Biotechnol. 2024 Jan 31;12:1339723. doi: 10.3389/fbioe.2024.1339723 (PMC10865367; doi:10.3389/fbioe.2024.1339723)
Supplement: Supplementary file 1 [file DataSheet2.PDF]

Supplementary File 2

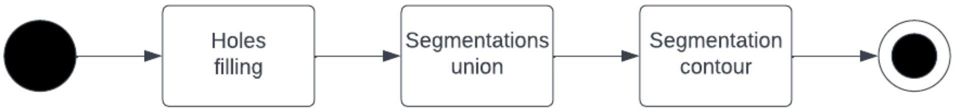

Supplementary Figure 2.1: Flowchart of "*Largest*" algorithm.

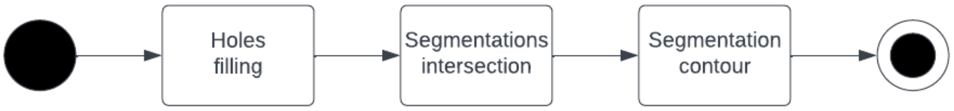

Supplementary Figure 2.2: Flowchart of "*Smallest*" algorithm.

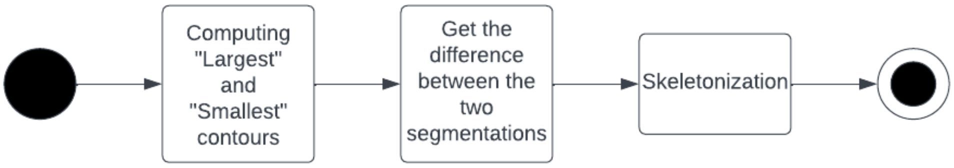

Supplementary Figure 2.3: Flowchart of "*Average Smallest And Largest*" algorithm.

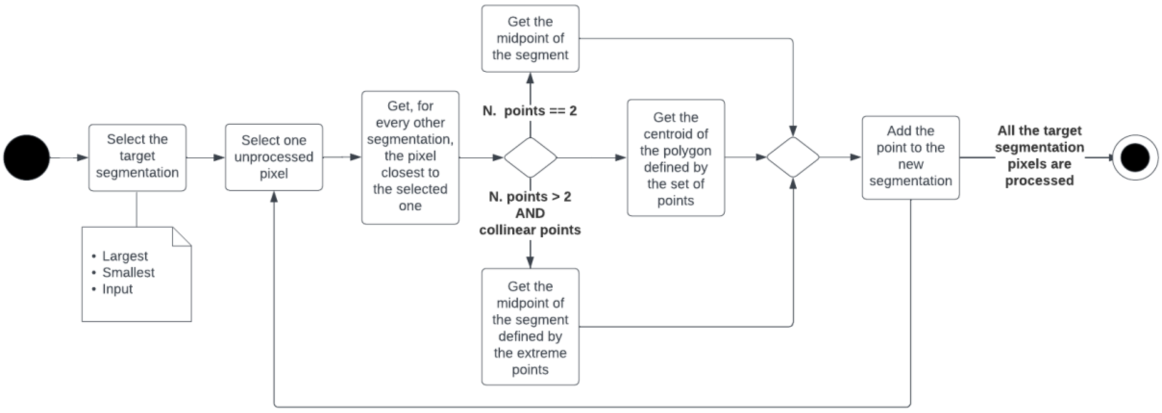

Supplementary Figure 2.4: Flowchart of "*Average With Target*" algorithms.

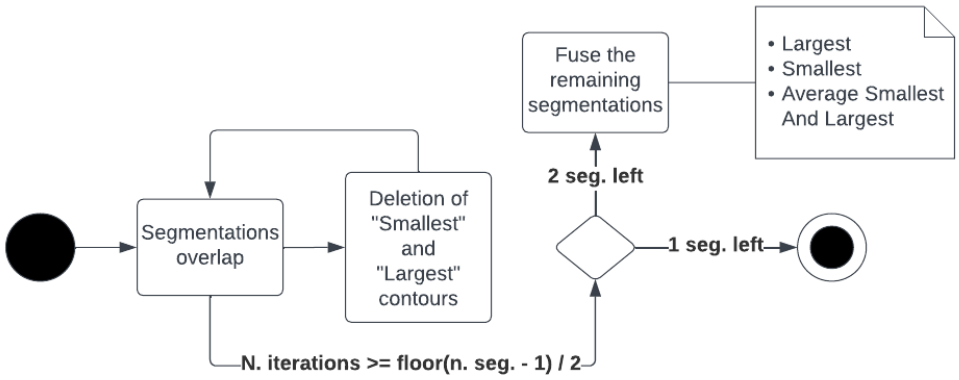

Supplementary Figure 2.5: Flowchart of "*Middle*" algorithm.

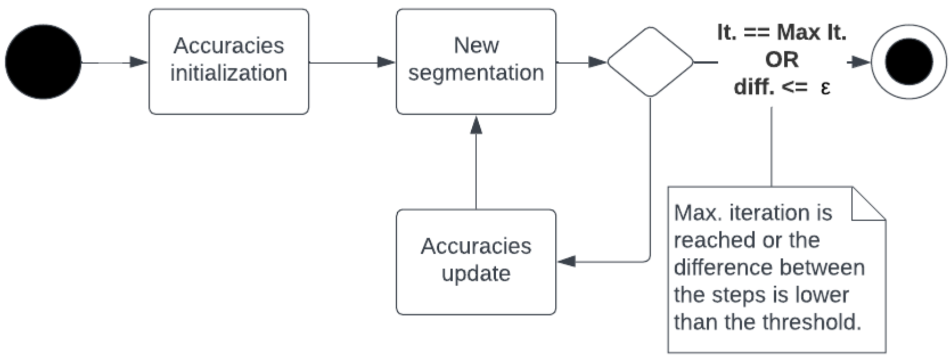

Supplementary Figure 2.6: Flowchart of "*STAPLE*" algorithm.
