# Supplementary figures and images for: Two-dimensional segmentation fusion tool: an extensible, free-to-use, user-friendly tool for combining different bidimensional segmentations
Source: Front Bioeng Biotechnol. 2024 Jan 31;12:1339723. doi: 10.3389/fbioe.2024.1339723 (PMC10865367; doi:10.3389/fbioe.2024.1339723)

# Supplementary File 1

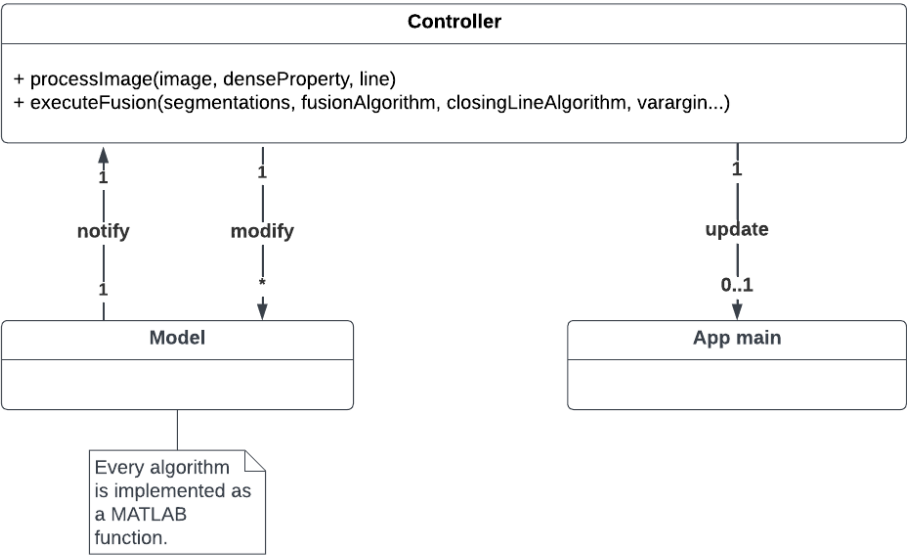

**Supplementary Figure 1.1:** TDSFT MVC architecture.

Supplement: Supplementary file 2 [file DataSheet1.PDF]
